# Principles of Surgery

**Published:** 1891-10

**Authors:** 


PRINCIPLES OF SURGERY. By N. SENN, M. D., Ph. D., Professor Principles of Surgery and Surgical Pathology in the Rush Medical College, Chicago, Ill.; Professor of Surgery in the Chicago Polyclinic; Attending Surgeon to the Milwaukee Hospital, etc., etc., etc. Illustrated with 109 wood engravings. Octavo, pp. xiii.—611. Philadelphia and London: F. A. Davis. 1890. Price, cloth, $4.50; sheep, $5.50 net.

This work, as its title indicates, is a treatise on the *principles* of surgery, pure and simple, and it must be so regarded and accepted in any criticisms that may be offered *pro* or *con* by its reviewers.

The author says in his preface: "It has been my aim to write a book for the student and general practitioner, which should, at least in part, fill this gap in surgical literature, and which should serve the purpose of a systematic treatise on the causation, pathology, diagnosis, prognosis, and treatment of the injuries and affections which the surgeon is most frequently called upon to treat."

It is a work based largely on the personal experience and study of the author, who fortunately has a rich mine of the former to draw upon, and a logical, studious brain to formulate and exemplify deductions therefrom for presentation to his readers.

The twenty-four chapters of the book bristle with original research, and teem with valuable thoughts, that are presented in an instructive and readable form. Senn is a clear writer, whose sentences are never involved, and hence he is not likely to be misunderstood. As might be expected in a modern work, written by a modern man, considerable prominence is given throughout the treatise to the subject of bacteriology. Chapter V., on Pathogenic Bacteria, is one of the best expositions we have seen of this intricate and many-sided question. A careful reading of this chapter will repay even the most astute bacteriologist, while it is so simple in its verbiage, and so methodical in its arrangement, as to attract the tyros in this field of study. The four chapters on Necrosis and Suppuration are equally simple, direct, instructive and masterly. And so we might go on through the volume, where we find so much to praise and so little to condemn, but we should weary the reader with tedious homilies, or consume his time with verbose iterations. This is a book that must be read to be appreciated, and we shall feel content if we have said anything that will stimulate even a few to possess it and study it for themselves.

It is printed in clear letter, on dull finished paper, which makes it pleasant reading for the eyes, while its matter entertains the mind. Its illustrations are chiefly in Pathology and Bacteriology, and help to give a completer understanding of the subjects which they elucidate.

The work has already become a standard text-book in the colleges, and will long continue to be quoted by teachers as the first and foremost authority on the principles of surgery.

